# Long-Term Care Market Trend and Patterns of Caregiving in the United States

**DOI:** 10.1093/geroni/igaa057.278

**Published:** 2020-12-16

**Authors:** Rashmita Basu

**Affiliations:** East Carolina University, Greenville, North Carolina, United States

## Abstract

Objectives: The current study aims to: 1) identify patterns of the use of long-term care services and supports (LTSS) among community-dwelling individuals; 2) examine if the changes in supply of formal care predict the use of informal care (IC). Methods: Linking the market supply of formal LTSS to individual level Health and Retirement Survey data from (N=7,781), descriptive and regression analysis were performed. Results: Supply of formal home and residential LTSS indicates a stronger upward trend. More than 90% of people used IC and 40% received both informal and formal help. People aged under 60 years, IC from spouse dominates but then falls sharply and an adult child becomes the primary source. More than 20% reported unmet needs. Regression analysis indicates that the formal home care supply significantly predicts the likelihood of using IC. But the rate and intensity of unpaid IC use among individuals aged 85 or older is low and that of paid formal care use are highest. We find that about 20% of care recipients experienced at least one unmet need with ADL assistance in our sample. The prevalence of an unmet need sharply decreases as individuals receive care from multiple caregivers (including paid professionals) but receiving care from too many caregivers contributes to higher unmet ADL needs. Discussion: The findings suggest opportunities to create a holistic system of care for people needing LTSS.

